# On the Efficiency of Haptic Based Object Identification: Determining Where to Grasp to Get the Most Distinguishing Information

**DOI:** 10.3389/frobt.2021.686490

**Published:** 2021-07-29

**Authors:** Yu Xia, Alireza Mohammadi, Ying Tan, Bernard Chen, Peter Choong, Denny Oetomo

**Affiliations:** ^1^ Human Robotics Laboratory, Department of Mechanical Engineering, The University of Melbourne, Parkville, VIC, Australia; ^2^ Department of Surgery, St. Vincent’s Hospital, The University of Melbourne, Parkville, VIC, Australia; ^3^ Aikenhead Centre for Medical Discovery (ACMD), St. Vincent’s Hospital, Parkville, VIC, Australia

**Keywords:** haptic based object identification, identification efficiency, object description, clustering, information gain

## Abstract

Haptic perception is one of the key modalities in obtaining physical information of objects and in object identification. Most existing literature focused on improving the accuracy of identification algorithms with less attention paid to the efficiency. This work aims to investigate the efficiency of haptic object identification to reduce the number of grasps required to correctly identify an object out of a given object set. Thus, in a case where multiple grasps are required to characterise an object, the proposed algorithm seeks to determine where the next grasp should be on the object to obtain the most amount of distinguishing information. As such, the paper proposes the construction of the object description that preserves the association of the spatial information and the haptic information on the object. A clustering technique is employed both to construct the description of the object in a data set and for the identification process. An information gain (IG) based method is then employed to determine which pose would yield the most distinguishing information among the remaining possible candidates in the object set to improve the efficiency of the identification process. This proposed algorithm is validated experimentally. A Reflex TakkTile robotic hand with integrated joint displacement and tactile sensors is used to perform both the data collection for the dataset and the object identification procedure. The proposed IG approach was found to require a significantly lower number of grasps to identify the objects compared to a baseline approach where the decision was made by random choice of grasps.

## 1 Introduction

Haptics is one of the important sensing modalities used to perceive object physical properties, surface properties, and interaction forces between the end-effectors and the objects (Shaw Cortez et al., 2019; Scimeca et al., 2020; Mayer et al., 2020). Object identification is one of the most important applications of haptics, particularly, in cases where the identification process needs to rely on the information provided only through the physical interaction between the end-effector and the objects, or when it cannot be conveniently achieved by other means (Dargahi and Najarian, 2004). While haptics is most likely used in conjunction with other sensing modalities in practice (e.g. with vision) (Liu et al., 2017; Faragasso et al., 2018), it is also important for haptic-based object identification to be studied in isolation to understand the extent of its capabilities. It should be noted that haptics refers to the description of an object through all the information obtained by touching the object (Overvliet et al., 2008; Grunwald, 2008; Hannaford and Okamura, 2016). In this work, the haptic information involves not only the tactile information at the contact points/surface but also the proprioceptive information such as the pose of the fingers upon touching the objects.

Accuracy and efficiency are two important evaluation metrics in object identification in general as well as in haptic object identification. In haptic-based object identification, multiple grasps of the object presented are often required to identify it from the given set of objects. This is also naturally observed in human efforts of object identification when relying on handling the object without vision (Gu et al., 2016; Luo et al., 2019). The efficiency of the identification process is therefore to do with identifying the object in as few numbers of grasps as possible. Most existing literature focuses on improving the accuracy. Examples include histogram-based methods (Luo et al., 2015; Schneider et al., 2009; Pezzementi et al., 2011; Zhang et al., 2016) and various supervised learning techniques (such as random forest and neural network) (Spiers et al., 2016; Schmitz et al., 2015; Liu et al., 2016; Funabashi et al., 2018; Mohammadi et al., 2019). Relative to the accuracy, the efficiency of haptic object identification techniques has been less investigated with only a few reported studies (Kaboli et al., 2019; Xu et al., 2013). In (Xu et al., 2013), an efficient exploratory algorithm was presented specifically for texture identification. In (Kaboli et al., 2019), a method was presented to improve the efficiency of the learning process of object physical properties for the purpose of constructing object descriptions, not object identification.

The objective of this paper is to provide a systematic method for analysing and quantifying the efficiency of haptic object identification. The process affects both stages of the exercise: the object dataset construction process and haptic object identification process based on information gain technique. An approach for the object dataset construction is proposed to preserve the association of the spatial information on the objects and the haptic information they yield. During the data set construction, the clustering technique is employed to reduce the dimensionality and complexity of the object descriptions, such as in (Pezzementi et al., 2011). Based on the preserved spatial information (haptic object description), the haptic information associated with each pose on the object is available to the algorithm. In contrast, histogram-based approaches (Luo et al., 2015; Schneider et al., 2009; Pezzementi et al., 2011; Zhang et al., 2016) construct object descriptions by counting how many times each grasp cluster appears in each object, but not which poses they belong to, thus cannot be utilised to determine where to grasp the objects to obtain specific information.

The proposed object label construction in the object dataset is then combined with a proposed information gain based technique to calculate the amount of information at each pose that can be obtained to distinguish the object to be identified from the rest of the objects in the object set. Efficiency in haptic object identification is pursued in this algorithm by selecting the pose that contains the largest amount of information. This leads to a significant reduction in the number of grasps required to correctly identify the object out of a given object set compared to the conventional practice where such object information is not utilised in the identification process.

## 2 Proposed Methodology

**FIGURE 1:**
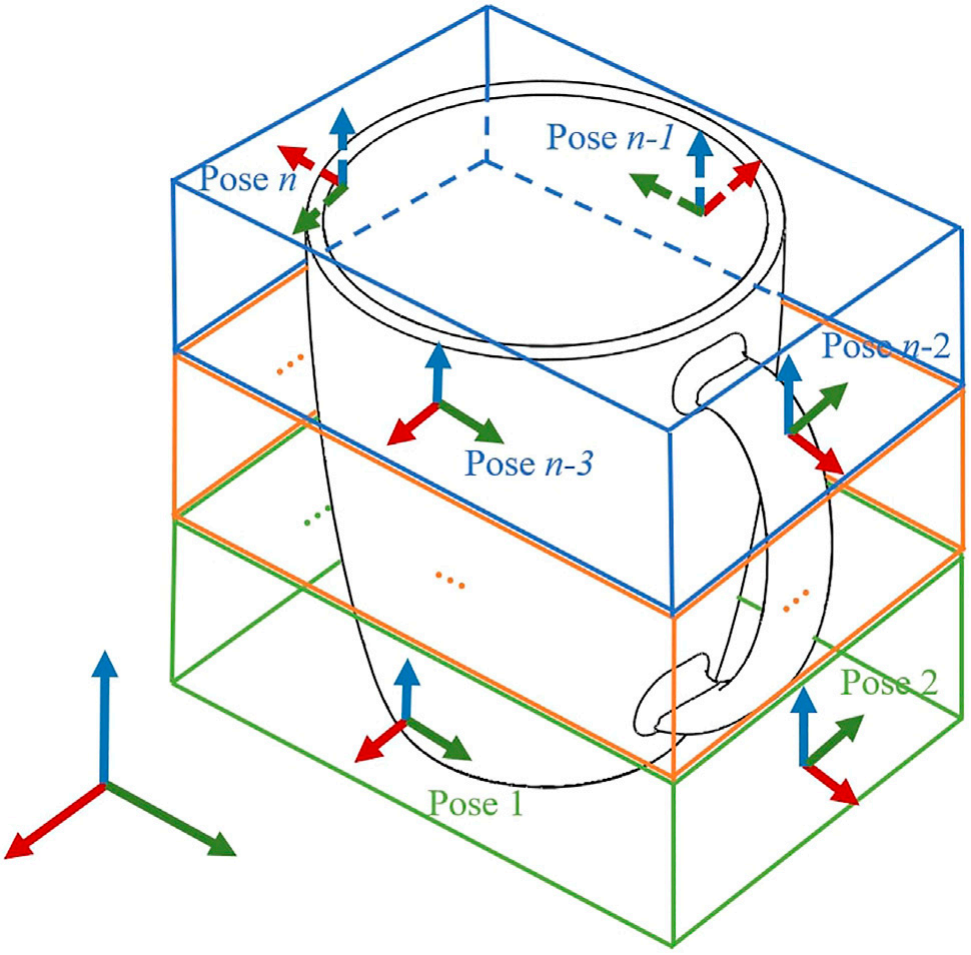
The characteristics of an object are regarded to be fully captured by grasping the object at *n* different poses. The *n* poses are spread, if possible uniformly, across the surface of the object.

Given a set of objects $O\;=\left\{o_{1},\ldots ,o_{l}\right\},\;\;l\in N$, where N is the set of integers, and a set of poses $P\;=\left\{p_{1},\ldots ,p_{n}\right\},\;\;n\in N$, it is assumed that for grasping each object *o*_*i*_ ∈ *O* (where i≤ l, i∈N), at all *n* poses is sufficient to fully capture its characteristics and thus uniquely define the object. Figure 1 shows an object with coordinate frames 1 to *n* representing the finite number of predefined poses where a robotic grasper with relevant tactile and proprioceptive sensors is placed to grasp the objects to gather measurements. These poses are specified only as the angle of approach to the object at each increment in object height (the example in Figure 1 shows three levels of object heights and the poses defined at every *π*/2 *rad* for each height). These predefined poses are consistent across all objects in the object set and assumed to be known *a priori*.

The objective of this work, therefore, is to identify an object presented to the algorithm, among possible objects in the object dataset, with the fewest number of grasps, where the possible grasp poses (the location and orientation that a robotic hand is used to grasp the object, relative to the object) are common for all objects.

The process involved in this exercise includes the construction of the object dataset (that incorporates the grasp pose information to the corresponding haptic information) and the object identification. Once the object dataset is established according to the proposed method, the problem is to identify which object (out of the object set) is being presented to the algorithm.

### 2.1 Object Dataset Construction

The object dataset construction procedure is presented in three steps: Data acquisition and normalisation, categorising the grasp clusters and, constructing the object description. The details of each step are presented below.

#### 2.1.1 Data Acquisition and Normalisation

Haptic measurements for all objects are taken at the predefined poses and the signals are processed. Each grasp on the object will yield a *M* dimensional measurement, corresponding to the number of sensors on the robotic hand. To collect the data, the robotic hand grasps each object at *n* different poses covering the object for all *ℓ* objects in the object set. The grasp at each pose is repeated *T* times to account for the expected amount of uncertainty associated with the grasping process, for example, the sensor noise and the robot hand pose uncertainties. Due to the different dimensions associated with the different types of physical variables involved (joint displacement and pressure), a normalisation method is needed to remove the influence of the dimension and the unit. In this work, the “Min-Max” normalisation method (Jain et al., 2005), which scales the values of all variables to the range of zero and one, is employed.

#### 2.1.2 Categorising the Grasp Clusters

An unsupervised learning technique is then utilised to cluster all the *M* dimensional normalised measurements for all *n* poses in an object for all objects in the dataset into *K* grasp clusters. In this paper, the well-established K-Means approach is adopted due to its high-speed performance. Details of the K-Means algorithm can be found in (Parsian, 2015). To determine the number of clusters, the ‘elbow method’ is used (Bholowalia and Kumar, 2014).

**FIGURE 2:**
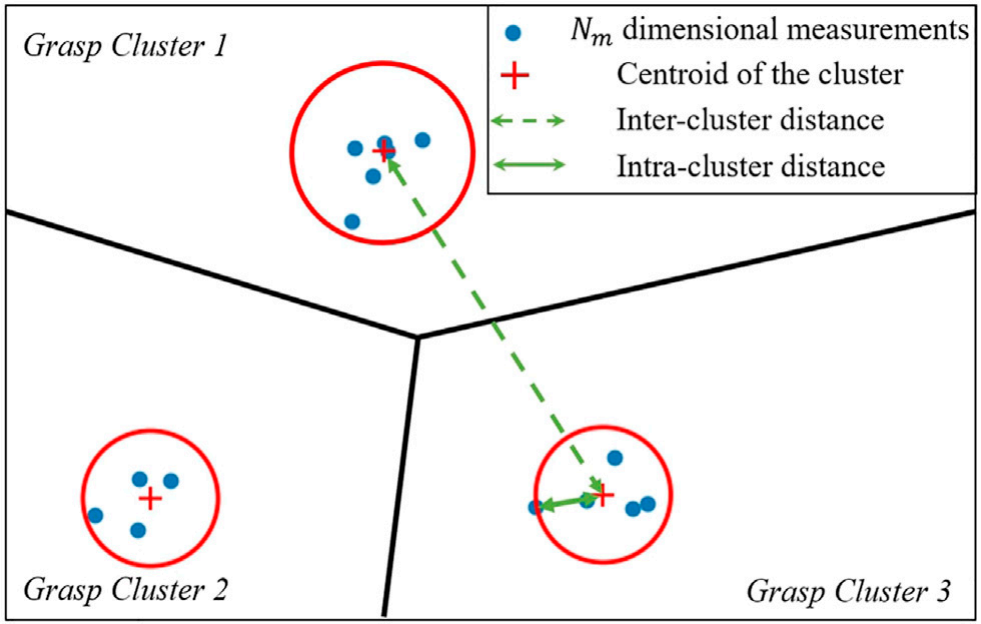
An illustration of a 2D projection of the clusters formed by the *M*-dimensional normalised measurements (represented by each point on the plot) for all *n* poses in an objects for all *ℓ* objects in the dataset. The points are categorised into *K* grasp clusters.

As an illustrative example, a 2-dimensional plot representing the clustering results is shown in Figure 2. Each point on Figure 2 represents 1 *M* dimensional normalised measurement (for ease of reading, the *M* dimension plot is conceptually represented in a 2-dimensional plot). Therefore, in order to focus the study on the efficiency of the algorithm, it is assumed that the object dataset is well designed with distinguishable objects, and noise/uncertainty in the haptic measurements are bounded in a range that is clearly smaller than the variance in the distinguishing information between distinct grasps. Therefore, grasping an object at a given pose repeated multiple times will result in measurements that should all be categorized into the same cluster, which guarantees the identification accuracy can be decoupled from the efficiency. Furthermore, for each cluster, the spread of measurements will be completely bounded (represented by the red circles in Figure 2) and the minimum distance between the centroids of distinct clusters (inter-cluster distance) should be much larger than the radius of this bound (intra-cluster distance). Note that this assumption is only used for the object dataset construction. During the object identification step, practical uncertainties are included in the test, where the effect of such uncertainties is evaluated in the experiment.

#### 2.1.3 Constructing the Object Description

**FIGURE 3:**
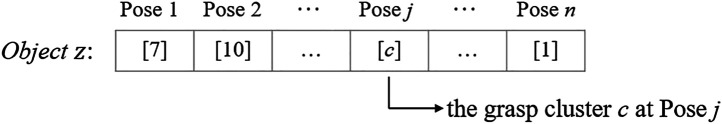
The description of an arbitrary object in the object set.

After categorisation, an object can be labelled uniquely by the string (of length *n*) for the *n* poses across the object. Each pose *p*_1_ … *p*_*n*_ is assigned a cluster number, which is the cluster that the haptic measurements at that pose for that object have been categorised into. An example of the label of an object in the set is represented in Figure 3. In this example, when grasped at Pose 1, the object yields haptic measurements across the *M* sensors on the robotic grasper that has been categorised into grasp cluster number seven through the clustering process. The resulting object description therefore preserves the information of which pose yields which grasp measurement, which is now represented as the grasp cluster number.

### 2.2 Object Identification

The proposed object identification approach in this paper utilises the information gain based (IG-based) technique. In this paper, it would be compared to a baseline approach, where the knowledge of the characteristics in the object dataset is not utilised. In this work, any objects presented to be identified should belong to the object set. If a new object (which does not belong to the object set) is presented, it should first be added to the object data set, by undergoing the Object Dataset Construction step.

It should be noted that when performing the object identification, the relative pose between the robotic grasper and the object is assumed to be known within the bound of uncertainties. This is a common assumption made in the studies of haptic-based object identification techniques, such as in (Madry et al., 2014; Luo et al., 2015; Schmitz et al., 2015; Liu et al., 2016; Spiers et al., 2016), to simplify the analysis and to keep the paper focused on the main question at hand. In practice, the relative displacements of subsequent poses grasped by the robotic end-effector are known to the algorithm that commands the robot. Only the pose of the initial grasp (the first grasp the robot makes contact with the object) is unknown. This, however, can be obtained as the robot makes its subsequent grasps, in a technique analogous to the well established simultaneous localisation and map building (SLAM). This technique, adapted to the haptic-based object identification problem, is established in the literature and was presented in (Lepora et al., 2013).

#### 2.2.1 Information Gain Based Approach (The Proposed Approach)

Information gain is a commonly used concept to represent the amount of information that is gained by knowing the value of a feature (variable) (Gray, 2011). Specifically, IG assigns the most distinguishable feature with the highest information value. Currently, IG has been developed and applied to decision trees, simultaneous localization and mapping (SLAM), and feature selection techniques (Kent, 1983; Lee and Lee, 2006) and in object shape re-construction by improving the exploration efficiency (Ottenhaus et al., 2018).

##### 2.2.1.1 Background on Information Gain Approach

For a random variable *X* with *a* possible outcomes, *x*_*α*_, *α* = 1, … , *a*, assume that each outcome has a probability of *p*(*x*_*α*_). The *information entropy H*(*X*) is defined as:

$$H\left(X\right)\;=-\sum_{\alpha =1}^{a}p\left(x_{a}\right)\log_{2}\left(\;p\left(x_{a}\right)\right).$$ (1)

*Conditional entropy* is then used to quantify the information needed to describe the outcome of a random variable *X* given the value of another random variable *Y*. Assume that there are *b* possible outcomes of the random variable *Y*. Each outcome *y*_*β*_ of the random variable *Y* has a probability of *p*(*y*_*β*_), *β* = 1, … , *b*. Let *p*(*x*_*α*_|*y*_*β*_) be the conditional probability of *x*_*α*_ given *y*_*β*_. *H*(*X*|*Y*) represents the information entropy of the variable *X* conditioned upon the random variable *Y*, which can be computed as:

$$\begin{array}{l} H\left(X\text{|Y}\right)\;=\sum_{\beta =1}^{b}p\left(y_{\beta }\right)H\left(X\text{|}y_{\beta }\right)\\ \;\;\;\;\;\;\;\;\;\;\;\;\;\;\;\;\;\;\;=-\sum_{\beta =1}^{b}p\left(y_{\beta }\right)\sum_{\alpha =1}^{a}p\left(x_{\alpha }\text{|}y_{\beta }\right)\log_{2}\left(p\left(x_{\alpha }\text{|}y_{\beta }\right)\right) \end{array}$$ (2)

*Information Gain* represents the degree to which uncertainty in the information is reduced when the random variable *Y* happens, which is defined as

G(X,Y)= H(X)−H(X|Y). (3)

##### 2.2.1.2 IG-Based Approach for Haptic Object Identification

An IG-based approach is proposed here that estimates the degree to which the information uncertainty is reduced by each choice of the pose in the haptic identification applied to a set of *ℓ* candidate objects.

At each iteration, the algorithm will calculate the information gain for each pose for every remaining object in the set. Firstly, the information entropy is calculated for each object. Considering that *N* remaining objects in the set, the given object will be sequentially indexed as *o*_1_, *o*_2_, … , *o*_*N*_. Note that at the first iteration, *N* = *ℓ*. When the object to be identified is assumed to be *o*_*i*_, all other remaining objects in the set will then be named *¬o*_*i*_ (i.e., not *o*_*i*_). Therefore, the object set for object *o*_*i*_ can be represented as two possible outcomes: O_oi_ = {o_i_, ¬o_i_}, representing the random variable. The probability of each possible outcome can then be calculated as:

$$p\left(o_{i}\right)\;=\frac{1}{N};$$ (4)

$$p\left(\neg \;\;o_{i}\right)\;=\frac{N-1}{N}.$$ (5)

Therefore, the information entropy for the object *o*_*i*_ can be calculated as:

$$H\left(O_{oi}\right)\;=-p\left(o_{i}\right)\log_{2}\left(P\left(o_{i}\right)\right)-p\left(\neg \;\;o_{i}\right)\log_{2}\left(p\left(\neg \;\;o_{i}\right)\right).$$ (6)

A second variable considered here is C_pj_ = {c_pj_,1,...,c_pj,gi_} containing non-repeating grasp cluster numbers at pose *p*_*j*_ for all remaining objects in the set, while *g*_*j*_ is the number of non-repeating grasp cluster numbers in the set C_pj_. At pose *p*_*j*_, each possible outcome $c_{pj,t}\in C_{pj}$ (where $t\leq g_{j},t\in N$) has a probability p (c_pj,t_). Defining a set D_pj_ = {d_pj_, _1_, ...,d_pj,gj_} to represent the frequency of occurrence of each non-repeating grasp cluster number c_pj,t_ at pose *p*_*j*_ (for example, at pose *p*_*j*_, the occurrence frequency of c_pj,1_ is d_pj,1_), the probability of the outcome c_pj,t_ can be calculated as:

$$p\left(c_{pj,t}\right)\;=\frac{d_{pj,t}}{N}.$$ (7)

Therefore, the conditional entropy for object *o*_*i*_ at pose *p*_*j*_ can be computed as

$$\begin{array}{l} H\left(O_{oi}\text{|}C_{pj}\right)\;=\sum_{t=1}^{N_{pj}}p\left(c_{pj,t}\right)H\left(O_{oi}\text{|}C_{pj,t}\right)\;\\ \;\;\;\;\;\;\;\;\;\;\;\;\;\;\;\;\;\;\;\;\;\;\;\;=-\sum_{t=1}^{N_{pj}}p\left(c_{pj,t}\right)\left[p\left(o_{i}\text{|}c_{pj,t}\right)\log_{2}\left(p\left(o_{i}\text{|}c_{pj,t}\right)\right)+p\left(\neg o_{i}\text{|}c_{pj,t}\right)\log_{2}\left(p\left(\neg o_{i}\text{|}c_{pj,t}\right)\right)\right]. \end{array}$$ (8)

The $p\left(o_{i}\text{|}c_{pj,t}\right)$ and $p\left(o_{i}\text{|}c_{pj,t}\right)$ are the conditional probability of *o*_*i*_ given c_pj,t_ and *¬o*_*i*_ given c_pj,t_, respectively. Specifically, if the grasp cluster number of *o*_*i*_ at *p*_*j*_ is c_pj,t_, the conditional probability can be calculated as:

$$p\left(o_{i}\text{|}c_{pj,t}\right)\;=\frac{1}{d_{pj,t}}.$$ (9)

$$p\left(\neg o_{i}\text{|}c_{pj,t}\right)\;=\frac{d_{pj,t-1}}{d_{pj,t}}.$$ (10)

otherwise, if the grasp cluster number of *o*_*i*_ at *p*_*j*_ is not c_pj,t_, then $p\left(o_{i}\text{|}c_{pj,t}\right)\;=0$ and $p\left(\neg o_{i}\text{|}c_{pj,t}\right)\;=1$.

The information gain (IG) for object *o*_*i*_ at pose *p*_*j*_ can be calculated as:

$$\begin{array}{l} G\left(o_{i},p_{j}\right)\;=H\left(O_{oi}\right)-H\left(O_{oi}\text{|}C_{pj}\right)\\ \;\;\;\;\;\;\;\;\;\;\;\;\;\;\;\;\;\;\;\;\;=-p\left(o_{i}\right)\log_{2}\left(p\left(o_{i}\right)\right)-p\left(\neg o_{i}\right)\log_{2}\left(p\left(\neg o_{i}\right)\right)\\ \;\;\;\;\;\;\;\;\;\;\;\;\;\;\;\;\;\;\;\;\;\;\;\;\;+\sum_{t=1}^{N_{pj}}p\left(c_{pj,t}\right)\left[p\left(o_{i}\text{|}c_{pj,t}\right)\log_{2}\left(p\left(o_{i}\text{|}c_{pj,t}\right)\right)+p\left(\neg o_{i}\text{|}c_{pj,t}\right)\log_{2}\left(p\left(\neg o_{i}\text{|}c_{pj,t}\right)\right)\right]. \end{array}$$ (11)

The information gain can be calculated for all remaining objects for each pose. The algorithm then selects the pose that contains the highest IG. In other words, the algorithm will grasp the object at the pose that would provide the most information gain. For each pose, each object has its IG value, representing the degree of distinction of this object from all other remaining objects in the set at this pose. At a given pose, the greater the IG value of an object, the greater the degree of distinction between this object and other remaining objects at this pose. There may be a few ways to quantify the measure of the “highest information gain”. In this paper, the algorithm first selects the pose with the largest value of minimum information gain (MIG). When the largest value of MIG is shared by more than one pose, the algorithm then chooses the pose with the highest value of average IG (AIG) out of these poses. If the highest value of AIG is yet shared by more than one pose, any of these poses can be chosen since they all have the same amount of information for the object identification purpose. If such situation arises, in this paper, the pose selection will follow the order of poses.

The summary of the proposed method is shown in Algorithm 1. Note that for a given object dataset, the first pose in the identification process is always the same, as the algorithm will go first for the pose with the largest value of minimum IG for the entire object dataset. After the first grasp, depending on the measurements encountered on the object to the identified, the appropriate candidate objects are eliminated from consideration and a “reduced object set” is generated, containing the remaining possible options (of objects) that could not be eliminated. The proposed algorithm will be repeated until the number of objects in the set is less than or equal to one. The outcome of the object identification process can be one of the following: 1) the object is correctly identified, or 2) the object is misidentified (as another in the object set). It is also possible in the case of misidentification that the resulting object description does not match any in the object dataset. In this case, 3) the object is considered as unidentified. Note that this does not imply that the new object is not contained in the object dataset. It simply means that the uncertainties in the measurements resulted in an object description that cannot be matched to the dataset.

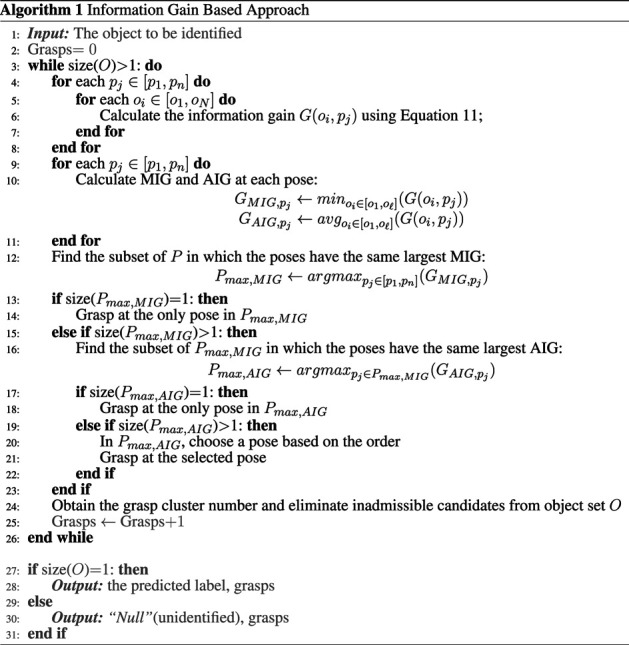

### 2.2.2 Baseline Approach

In this paper, a baseline approach is constructed to be compared with the proposed IG based approach, and the summary of it is shown in **Algorithm 2**. By baseline approach, we mean that the decision on where to grasp next in the haptic object identification process does not consider the information content of the object dataset. The procedure of the baseline approach is similar to the IG based approach (it still eliminates inadmissible candidates following the grasps performed), but the subsequent grasp pose is selected at random. The grasp poses where measurements are already taken are removed from the set *p*, which means this grasp pose can not be chosen in the following identification iterations. The baseline approach has also been utilised in (Corradi et al., 2015; Vezzani et al., 2016; Zhang et al., 2016) where efficiency is not the focus of their study.

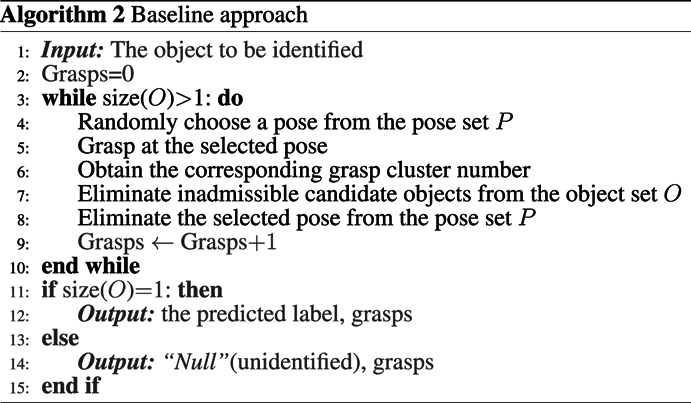

## 3 Experimental Evaluation

### 3.1 Experimental Setup

To evaluate the performance of the proposed object identification algorithm, the following experimental setup is used in this paper:

#### 3.1.1 Object Set

**FIGURE 4:**
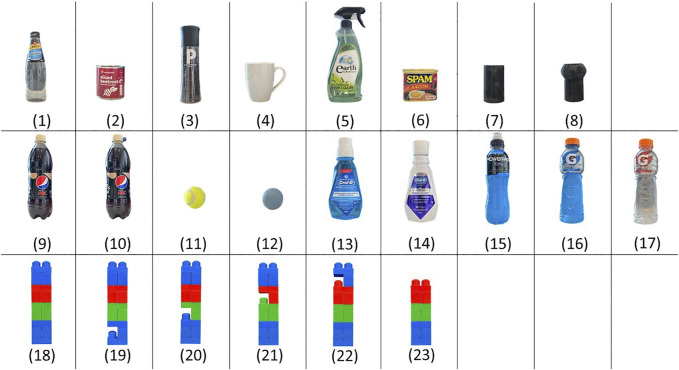
Consists of 23 objects in a variety of shapes, sizes and stiffness.

To represent a wide range of objects with different values of stiffness and shapes, 23 objects are selected in this study (as shown in Figure 4). Objects used for this study are: 1) Glass bottle, 2) Cylindrical can, 3) Peppercorn dispenser, 4) Mug, 5) Spray bottle, 6) Cuboid can, 7) 3D printed cylinder, 8) Arbitrarily shaped 3D-printed object, 9) Pepsi bottle, and 10) Pepsi bottle otherwise identical to Object 9) but with a bump on the bottle cap, 11) Tennis ball (soft), 12) Silicone ball (hard), 13) Oral-B blue bottle, 14) Oral-B white bottle, 15) Soft drink bottle, 16) Sports drink bottle (full), 17) Sports drink bottle (empty), 18) Assembled Lego blocks, 19) Assembled Lego blocks with a specific part at Height 1, 20) Assembled Lego blocks with a specific part at Height 2, 21) Assembled Lego blocks with a specific part at Height 3, 22) Assembled Lego block with a specific part at Height 4, 23) Assembled Lego blocks with a shorter height. All selected objects satisfy the assumption on distinguishability of the objects.

Objects in the set are intentionally selected to have a high level of similarity, such as Objects 9 and 10 which are identical except for the bump on the bottle cap on Object 10. Object 11 and 12 have the same shape of their main body, with stiffness difference overall. Object 13 and 14 have similar size and shapes but with different stiffness at Pose 1. Therefore, when grasping these two, the joint angle will be similar, but the tactile map will be different. Object 15 and 16 have similar object convex envelopes, such that they result in similar finger displacements when grasped. However, they differ in that one has clearly defined edges while the other shows a rounded shape. The tactile measurements upon grasping these two objects are significantly different, while the finger displacements of the grasps are similar. Object 15 and 17 are the same object (identical plastic bottles), but one is filled full with water while the other is empty. Object 18–23 have similar shapes on the majority of the portions of the objects, except on one specific part on each object.

#### 3.1.2 Robotic Hand and Tactile Sensors

**FIGURE 5:**
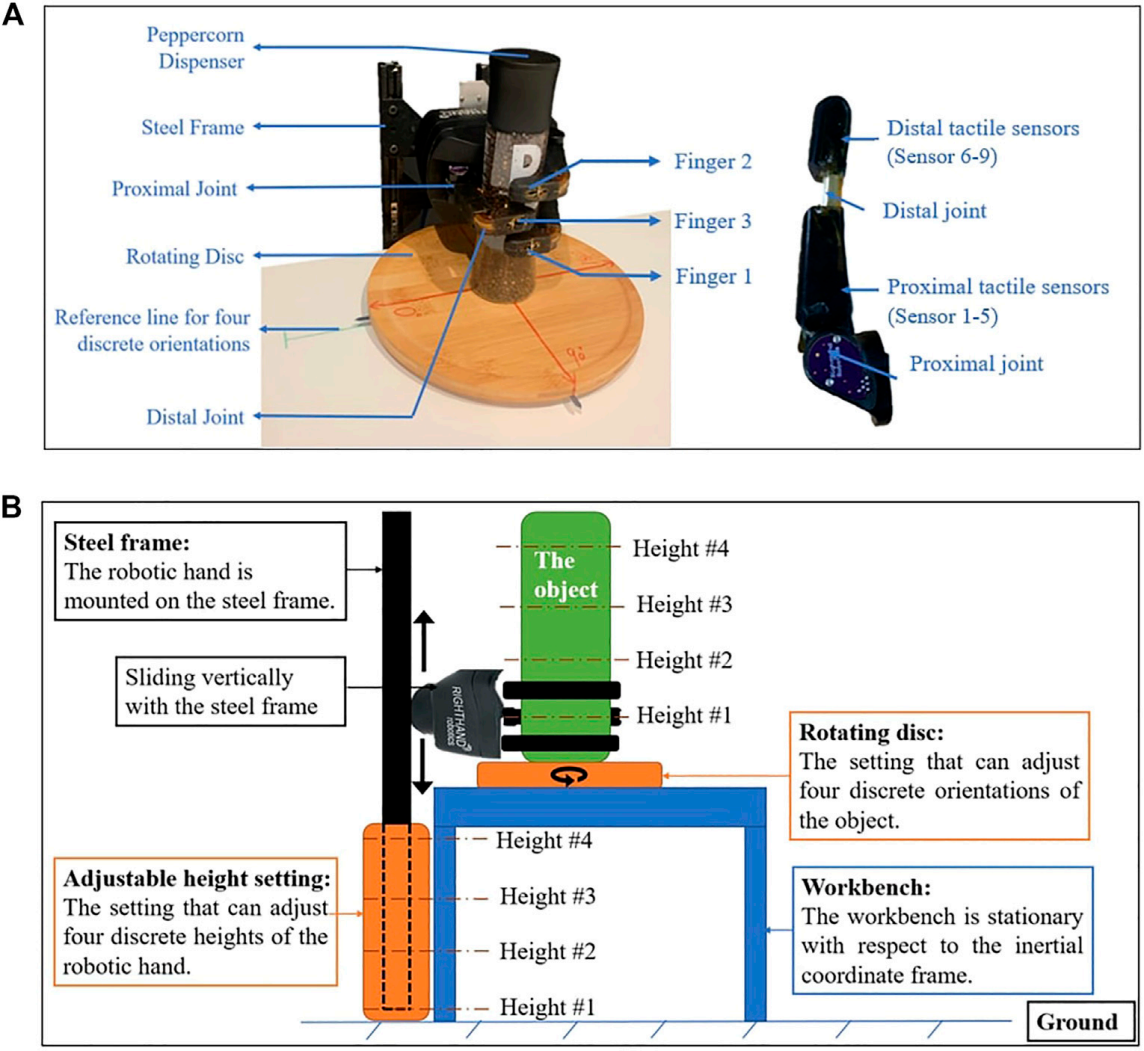
Experimental platform. **(A)** The physical experimental setup. **(B)** The schematic of the experimental setup (side view).

The experimental platform is shown in Figure 5. The ReFlex TakkTile (Right Hand Robotics, US), which consists of three under-actuated fingers, is used in the study. The ReFlex hand has 4 Degrees of Freedom (DoFs): the flexion/extension of each finger and one coupled rotation between the orientation of finger *No*.1 and *No*.2). In this work, only the three DoFs of finger flexion/extension are used. Each under-actuated finger is controlled by an actuator that can drive the tendon spanning both the proximal and distal joint. The proximal joint connects the proximal link to the base, and the distal joint connects the distal link to the proximal link. For each finger, there are one proximal joint encoder, one tendon spool encoder, and nine embedded Takktile pressure sensors arranged along with the finger. The reading of the tendon spool encoder represents the angular displacement of the whole finger (the sum of the angular displacement of proximal joint and distal joint), representing the proprioceptive information. Therefore, the degree of flexion of the distal joint can be calculated from the difference between the tendon spool decoder and the proximal joint encoder.

In this work, the ReFlex hand is mounted on a steel frame and its three fingers are positioned parallel to the horizontal direction. The height of the robotic hand can be determined by adjusting the vertical position of the steel frame. All the data is collected at 40*Hz* sampling. During each grasp, all fingers are commanded to move 3 *rad* over 6 s. A torque threshold is also set for each motor. For each finger, once this threshold is reached, this finger will stop moving and keep that pose as the final pose for measurements.

### 3.1.3 Software

Robotic hand operation and data collection are all performed via Robot Operating System (ROS), version ‘ROS-indigo’, under Linux Ubuntu 14.04 computer system. The information (joint angle and pressure value information) is recorded via ‘rosbag’ command and is saved in the ‘comma-separated values’(’.csv’) file.

### 3.2 Object Dataset Construction

Each object *o*_*ℓ*_ ∈ *O*, *ℓ* = 1, … , 23 is grasped at 16 different poses, *n* = 16, divided across four grasp heights (with 55 *mm* increments). The object is grasped in four different orientations about the vertical axis at each height setting (with 90^*o*^ increments). For each object, the grasp is repeated 10 times at each pose. The collected data are then normalised using the “Min-Max” normalisation method. To categorise the data, fifty-three grasp clusters are identified through the ‘elbow method’. Since each object is grasped at *n* = 16 different poses, the label for each object is made up of a series of *n* = 16 grasp cluster numbers.

### 3.3 Object Identification

Using the established data set, the object identification procedure with IG-based algorithm for efficiency was validated for two scenarios, where the measurements taken for the object identification process were done:

• **Scenario 1**: without uncertainty. This represents a sanity check, representing the theoretical-best-possible outcome, evaluating only the ability of the IG-based algorithm in utilising the information of the objects in the dataset to make decisions on where to make the subsequent measurement grasp. Note that the sensor measurement errors (of the robotic grasper encoders and tactile sensors) are still present in this case but were not significant to cause any false outcomes in identifying the grasp types.
• **Scenario 2**: with an amount of uncertainties approximated to a typical practical process. In this paper, the amount of practical uncertainties was realised as the positioning error of the grasper relative to the object, bounded within ±20 degrees of grasping orientation uncertainty (with respect to the object). This scenario evaluates the performance of the proposed method in a practical situation where measurement and grasp positioning noise are present.

Two tests are conducted in both scenarios: the accuracy evaluation and the efficiency evaluation. In the *accuracy evaluation*, ten sets of identification trials for each object are conducted, and the identification accuracy of each object will be recorded. The procedure is shown in Algorithm 3. In the *efficiency evaluation*, each object presented is to be correctly identified ten times. The procedure of the efficiency evaluation is summarised in Algorithm 4. The measure of evaluation is defined as the number of grasps used to correctly identify the presented object. When uncertainties are involved in the identification, where there is a possibility of the algorithm misidentify the grasp type when measuring one of the poses, this definition means that in each (of the ten attempts per object presented) to identify an object correctly, the number of grasps made on the object when a correct identification is not achieved are also counted towards calculating the efficiency. Each time the algorithm misidentify the presented object, the algorithm starts a new attempt by identifying the object from the beginning.

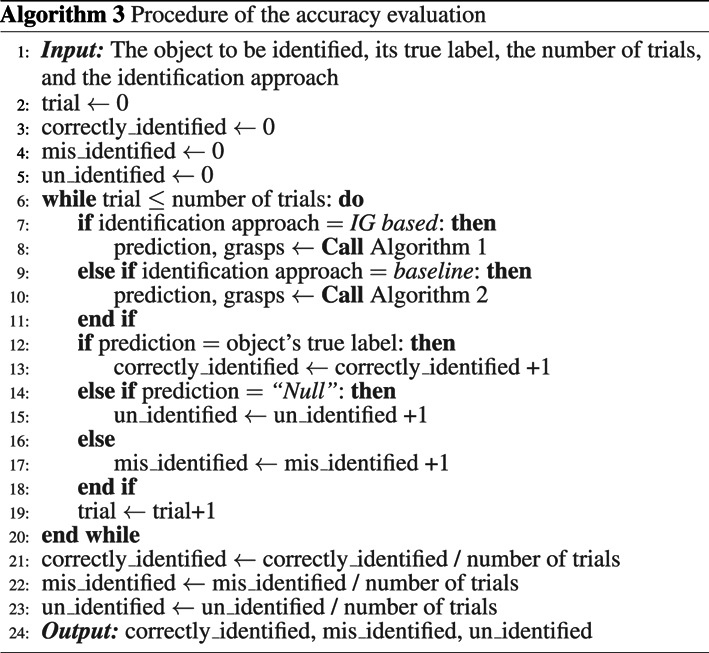

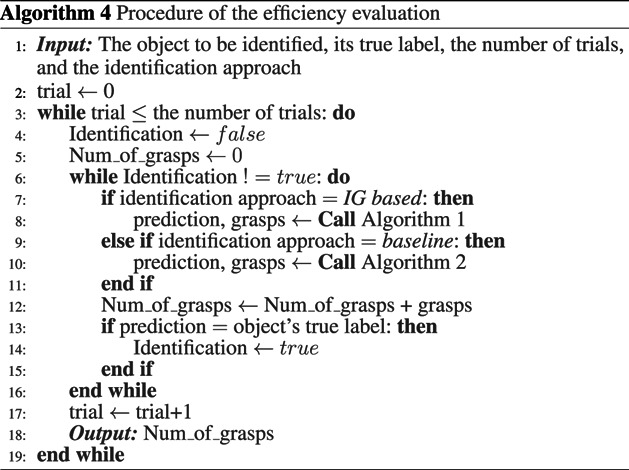

## 4 Results and Discussion

### 4.1 Object Dataset Construction

The result of the object dataset construction used in the experimental evaluation is represented in Table 1. In the resulting object dataset, each of the 23 objects is described as a 16-number long string, representing the 16 cluster numbers for that specific object from the measurements taken at the 16 grasping poses performed on the object.

**TABLE 1:** The list of the constructed labels for the object dataset used in the experiment.

	Pose 1	Pose 2	Pose 3	Pose 4	Pose 5	Pose 6	Pose 7	Pose 8	Pose 9	Pose 10	Pose 11	Pose 12	Pose 13	Pose 14	Pose 15	Pose 16
Object 1	(0)	(0)	(0)	(0)	(1)	(1)	(1)	(1)	(2)	(2)	(2)	(2)	(3)	(3)	(3)	(3)
Object 2	(4)	(4)	(4)	(4)	(5)	(5)	(5)	(5)	(3)	(3)	(3)	(3)	(3)	(3)	(3)	(3)
Object 3	(6)	(6)	(6)	(6)	(6)	(6)	(6)	(6)	(6)	(6)	(6)	(6)	(7)	(7)	(7)	(7)
Object 4	(8)	(8)	(8)	(9)	(10)	(11)	(12)	(13)	(3)	(3)	(3)	(3)	(3)	(3)	(3)	(3)
Object 5	(14)	(9)	(14)	(9)	(14)	(9)	(14)	(9)	(14)	(15)	(14)	(15)	(16)	(16)	(16)	(16)
Object 6	(17)	(9)	(17)	(9)	(3)	(3)	(3)	(3)	(3)	(3)	(3)	(3)	(3)	(3)	(3)	(3)
Object 7	(18)	(18)	(18]	(18)	(19)	(19)	(19)	(19)	(3)	(3)	(3)	(3)	(3)	(3)	(3)	(3)
Object 8	(18)	(18)	(18)	(18)	(20)	(20)	(20)	(20)	(3)	(3)	(3)	(3)	(3)	(3)	(3)	(3)
Object 9	(21)	(21)	(21)	(21)	(22)	(22)	(22)	(22)	(23)	(23)	(23)	(23)	(24)	(24)	(24)	(24)
Object 10	(21)	(21)	(21)	(21)	(22)	(22)	(22)	(22)	(23)	(23)	(23)	(23)	(25)	(24)	(24)	(24)
Object 11	(26)	(26)	(26)	(26)	(3)	(3)	(3)	(3)	(3)	(3)	(3)	(3)	(3)	(3)	(3)	(3)
Object 12	(27)	(27)	(27)	(27)	(3)	(3)	(3)	(3)	(3)	(3)	(3)	(3)	(3)	(3)	(3)	(3)
Object 13	(28)	(29)	(28)	(29)	(30)	(31)	(30)	(31)	(32)	(33)	(32)	(33)	(34)	(34)	(34)	(34)
Object 14	(35)	(29)	(35)	(29)	(30)	(31)	(30)	(31)	(32)	(33)	(32)	(33)	(36)	(36)	(36)	(36)
Object 15	(37)	(37)	(37)	(37)	(38)	(38)	(38)	(38)	(39)	(39)	(39)	(39)	(40)	(40)	(40)	(40)
Object 16	(39)	(41)	(39)	(41)	(42)	(43)	(42)	(43)	(44)	(44)	(44)	(44)	(45)	(45)	(45)	(45)
Object 17	(39)	(41)	(39)	(41)	(46)	(43)	(46)	(43)	(44)	(44)	(44)	(44)	(45)	(45)	(45)	(45)
Object 18	(47)	(48)	(47)	(48)	(47)	(48)	(47)	(48)	(47)	(48)	(47)	(48)	(47)	(48)	(47)	(48)
Object 19	(49)	(50)	(47)	(48)	(47)	(48)	(47)	(48)	(47)	(48)	(47)	(48)	(47)	(48)	(47)	(48)
Object 20	(47)	(48)	(47)	(48)	(49)	(50)	(47)	(48)	(47)	(48)	(47)	(48)	(47)	(48)	(47)	(48)
Object 21	(47)	(48)	(47)	(48)	(47)	(48)	(47)	(48)	(49)	(50)	(47)	(48)	(47)	(48)	(47)	(48)
Object 22	(47)	(48)	(47)	(48)	(47)	(48)	(47)	(48)	(47)	(48)	(47)	(48)	(49)	(50)	(47)	(48)
Object 23	(47)	(48)	(47)	(48)	(47)	(48)	(47)	(48)	(47)	(48)	(47)	(48)	(51)	(52)	(51)	(52)

### 4.2 Object Identification

#### 4.2.1 Scenario 1 (Without Uncertainties)

##### Accuracy Evaluation

Ten sets of identification trials for each object are conducted. In this scenario where no uncertainties were included, it was confirmed that all trials yielded a 100*%* accuracy for both the IG-based approach and the baseline approach, as expected.

##### Efficiency Evaluation

**FIGURE 6:**
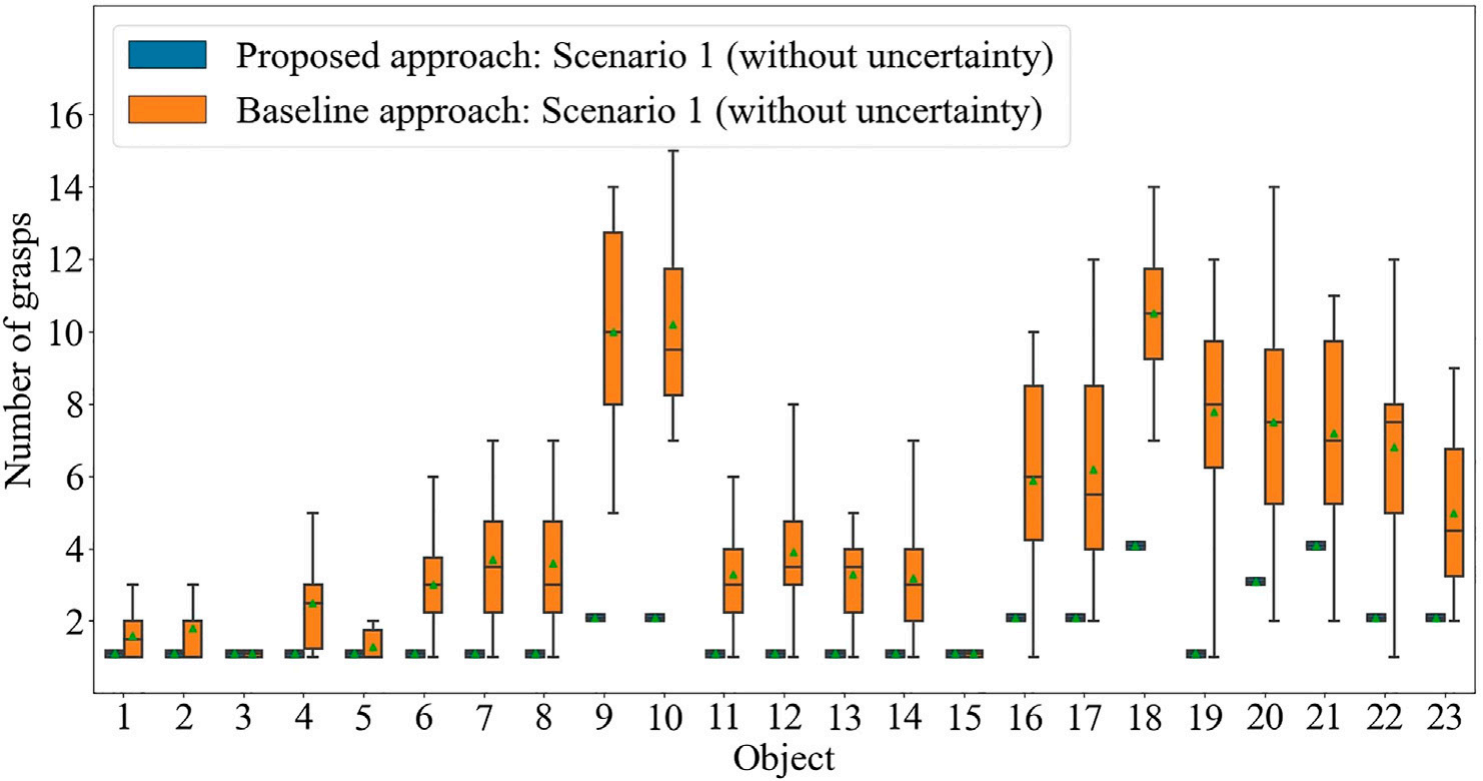
The number of grasps required to correctly identify each of 23 objects using the proposed approach and the baseline approach: Scenario 1 (without uncertainty).

The number of grasps required to correctly identify the presented object, using the baseline approach and the proposed IG-based approach, are shown in Figure 6. Using the constructed object dataset, the IG value for each object at each pose can be calculated for the first iteration. The IG values for all the objects for all possible poses are shown in Table 2. To decide on the pose for the first grasp, the algorithm looks at the largest minimum IG (MIG) values for all the poses in Table 2. In our example, a MIG value of 0.10 can be found for Pose 1 and 2, and Pose 5 and 6 (see the last row of Table 2). The algorithm then selects out of these poses, the one with the highest averaged IG (AIG) value, which is 0.20 for Pose 1. Therefore, for the first grasp, Pose 1 is selected. Note that once the first grasp is made, depending on the grasp type identified, inadmissible candidates are removed and the IG values for the subsequent grasp will need to be calculated with the set of the remaining objects.

**TABLE 2:** IG value for each object at each pose: first iteration.

	Pose 1	Pose 2	Pose 3	Pose 4	Pose 5	Pose 6	Pose 7	Pose 8	Pose 9	Pose 10	Pose 11	Pose 12	Pose 13	Pose 14	Pose 15	Pose 16
Object 1	0.26	0.26	0.26	0.26	0.26	0.26	0.26	0.26	0.26	0.26	0.26	0.26	0.07	0.07	0.07	0.07
Object 2	0.26	0.26	0.26	0.26	0.26	0.26	0.26	0.26	0.08	0.08	0.08	0.08	0.07	0.07	0.07	0.07
Object 3	0.26	0.26	0.26	0.26	0.26	0.26	0.26	0.26	0.26	0.26	0.26	0.26	0.26	0.26	0.26	0.26
Object 4	0.26	0.26	0.26	0.14	0.26	0.26	0.26	0.26	0.08	0.08	0.08	0.08	0.07	0.07	0.07	0.07
Object 5	0.26	0.17	0.26	0.14	0.26	0.26	0.26	0.26	0.26	0.26	0.26	0.26	0.26	0.26	0.26	0.26
Object 6	0.26	0.17	0.26	0.14	0.14	0.14	0.14	0.14	0.08	0.08	0.08	0.08	0.07	0.07	0.07	0.07
Object 7	0.17	0.17	0.17	0.17	0.26	0.26	0.26	0.26	0.08	0.08	0.08	0.08	0.07	0.07	0.07	0.07
Object 8	0.17	0.17	0.17	0.17	0.26	0.26	0.26	0.26	0.08	0.08	0.08	0.08	0.07	0.07	0.07	0.07
Object 9	0.17	0.17	0.17	0.17	0.17	0.17	0.17	0.17	0.17	0.17	0.17	0.17	0.26	0.17	0.17	0.17
Object 10	0.17	0.17	0.17	0.17	0.17	0.17	0.17	0.17	0.17	0.17	0.17	0.17	0.26	0.17	0.17	0.17
Object 11	0.26	0.26	0.26	0.26	0.14	0.14	0.14	0.14	0.08	0.08	0.08	0.08	0.07	0.07	0.07	0.07
Object 12	0.26	0.26	0.26	0.26	0.14	0.14	0.14	0.14	0.08	0.08	0.08	0.08	0.07	0.07	0.07	0.07
Object 13	0.26	0.17	0.26	0.17	0.17	0.17	0.17	0.17	0.17	0.17	0.17	0.17	0.26	0.26	0.26	0.26
Object 14	0.26	0.17	0.26	0.17	0.17	0.17	0.17	0.17	0.17	0.17	0.17	0.17	0.26	0.26	0.26	0.26
Object 15	0.26	0.26	0.26	0.26	0.26	0.26	0.26	0.26	0.26	0.26	0.26	0.26	0.26	0.26	0.26	0.26
Object 16	0.17	0.17	0.17	0.17	0.26	0.17	0.26	0.17	0.17	0.17	0.17	0.17	0.17	0.17	0.17	0.17
Object 17	0.17	0.17	0.17	0.17	0.26	0.17	0.26	0.17	0.17	0.17	0.17	0.17	0.17	0.17	0.17	0.17
Object 18	0.10	0.10	0.09	0.09	0.10	0.10	0.09	0.09	0.10	0.10	0.09	0.09	0.12	0.12	0.10	0.10
Object 19	0.26	0.26	0.09	0.09	0.10	0.10	0.09	0.09	0.10	0.10	0.09	0.09	0.12	0.12	0.10	0.10
Object 20	0.10	0.10	0.09	0.09	0.26	0.26	0.09	0.09	0.10	0.10	0.09	0.09	0.12	0.12	0.10	0.10
Object 21	0.10	0.10	0.09	0.09	0.10	0.10	0.09	0.09	0.26	0.26	0.09	0.09	0.12	0.12	0.10	0.10
Object 22	0.10	0.10	0.09	0.09	0.10	0.10	0.09	0.09	0.10	0.10	0.09	0.09	0.26	0.26	0.10	0.10
Object 23	0.10	0.10	0.09	0.09	0.10	0.10	0.09	0.09	0.10	0.10	0.09	0.09	0.26	0.26	0.26	0.26
AIG	0.20	0.19	0.19	0.17	0.19	0.19	0.18	0.18	0.15	0.15	0.14	0.14	0.16	0.15	0.14	0.14
MIG	0.10	0.10	0.09	0.09	0.10	0.10	0.09	0.09	0.08	0.08	0.08	0.08	0.07	0.07	0.07	0.07

The experiment results of the number of grasps required to correctly identify the object through the IG-based approach are shown in Figure 6. It is observed that the IG-based approach outperforms the baseline approach in general, even if the difference in performance differs from object to object. The average number of grasps needed for the baseline approach is 4.81 while the IG-based approach was observed to correctly identify the object on average in 1.65 grasps. As expected, the number of grasps required by the baseline approach to identify an object varies widely between different attempts due to the random selection in deciding the subsequent grasps. The IG-based approach is repeatable for all objects in the same set in the absence of practical uncertainties. This demonstrates the theoretically achievable performance of the proposed approach.

The advantage of the proposed approach is highlighted in the more challenging cases, where objects are more similar to others in the object set. Objects 9 and 10 are identical except for a dent in one of the two bottle caps - and the baseline approach struggled to identify the object without exploiting the knowledge of the objects in the dataset. Similar observations can be made to Objects 18 to 23 (See Figure 4).

#### 4.2.2 Scenario 2 (With Uncertainty)

This section explores how the proposed object identification approach performs when the measurement includes a level of uncertainty typical to that seen in practical conditions. The identification procedure remains the same as shown in Section 4.2.1 where the first grasp is carried out at Pose 1.

##### Accuracy Evaluation

**FIGURE 7:**
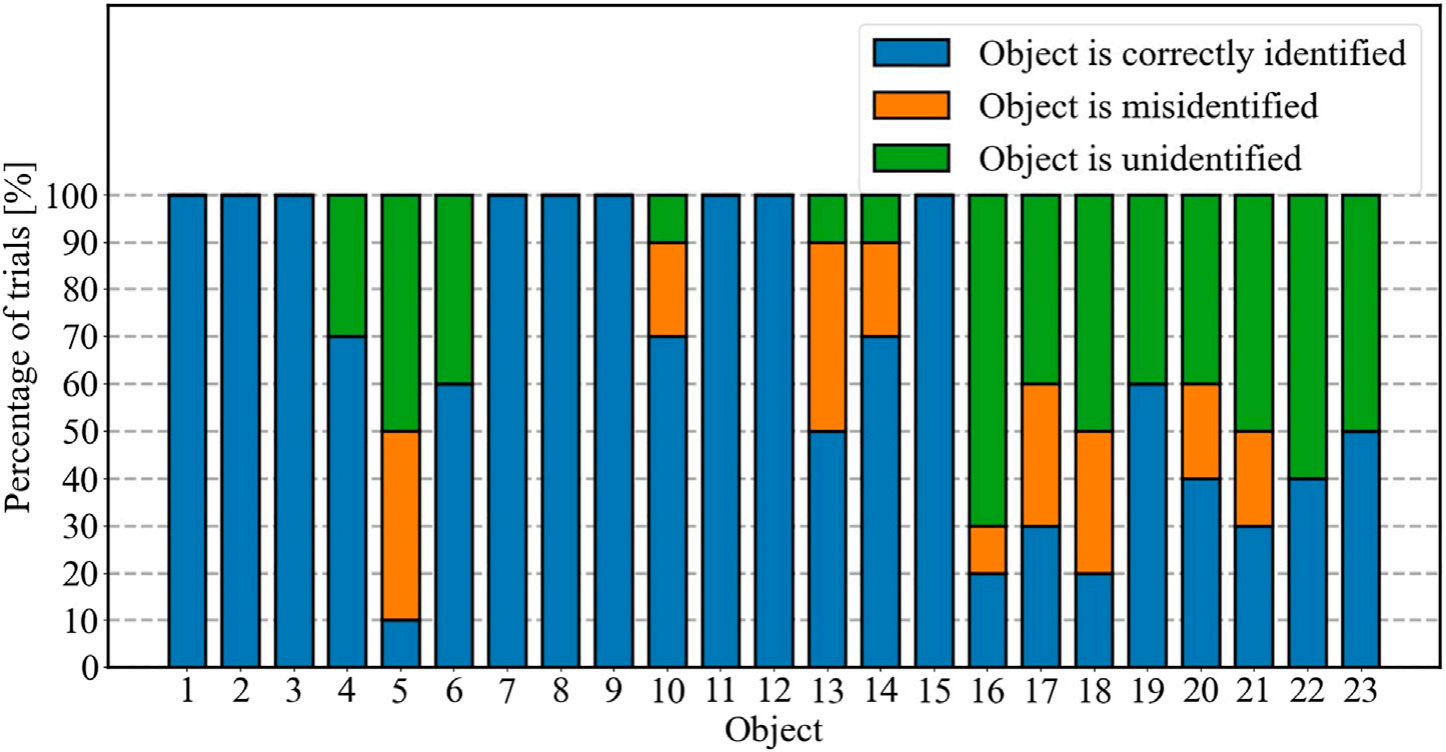
The accuracy of identifying each of 23 objects through the proposed approach: Scenario 2 (with uncertainty).

The resulting percentage accuracy of the proposed approach in the presence of practical uncertainties is shown in Figure 7. The percentage of misidentification (when an object identified as another) and unidentified (when the resulting descriptor does not match any object in the dataset due to the uncertainties involved) are also shown. It can be seen that some objects are more susceptible to uncertainties. In general, more cylindrical objects are less susceptible, while objects with sharp, angular features are more prone to misidentification. This is due to the nature of the tactile sensors on the robotic grasper used in our experiment, where individual sensors are embedded in the fingers. A change in the grasping pose will cause a sharp angular feature on an object to press on the adjacent tactile sensor, activating a completely different sensor in the resulting reading.

##### Efficiency Evaluation

**FIGURE 8:**
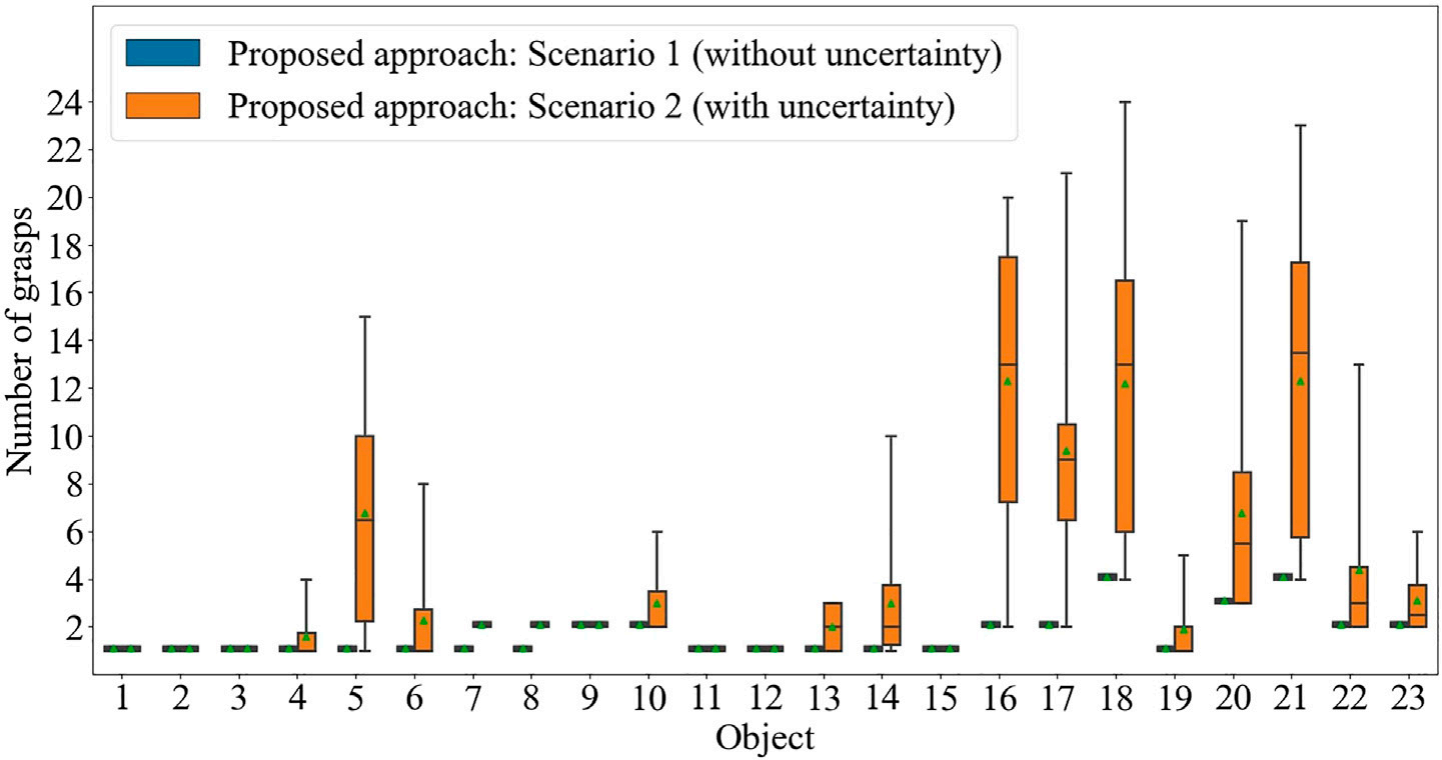
The number of grasps required to correctly identify each of 23 objects using the proposed approach: Scenario 2 (with uncertainty).

The effect of the inclusion of the practical uncertainties in the performance of the proposed approach is shown in Figure 8, where it is compared to the performance without practical uncertainties. The average number of grasps required for correct identification increases from 1.65 Scenario 1 to 4.08 in this Scenario.

As seen in the outcome of the accuracy evaluation, the identification efficiency of rectangular-shaped objects - objects with sharp angular features on their surface (such as Object 5, 6, 13, 14, and 18) are the most affected, due to its poor accuracy, thus requiring, on average, more attempts (thus more grasps) to make a correct identification. It is also demonstrated that the constructed measure of efficiency is successful in capturing/accounting for the accuracy of the identification approach in reflecting the efficiency performance of the approach.

## 5 Conclusion

This paper presents an approach to utilise the information in the object dataset in improving the efficiency of a haptic object identification approach. It is shown that compared to an equivalent haptic-based object identification approach that does not exploit such information (representative of the conventional approaches), the number of grasps required to correctly identify the presented object was significantly reduced. In the experimental evaluation with 23 objects in the object set, the proposed approach required on average 1.65 grasps to 4.81 grasps in the baseline approach. The presence of uncertainties in any practical applications, such as in the positioning inaccuracies of the pose of the robotic grasper upon the object or the sensor noise in haptic sensors onboard the robotic grasper, affects the performance of the approach. This is because inaccuracies in the identification approach require the algorithms more attempts to achieve the same number of correct identification of the objects. It was observed that the shapes of the objects and their interaction with the robotic grasper used in the task also determine the susceptibility of the object to these uncertainties.

## Data Availability

The raw data supporting the conclusion of this article will be made available by the authors, without undue reservation.
